# A Data-Driven Diagnostic Framework for Wind Turbine Structures: A Holistic Approach

**DOI:** 10.3390/s17040720

**Published:** 2017-03-30

**Authors:** Simona Bogoevska, Minas Spiridonakos, Eleni Chatzi, Elena Dumova-Jovanoska, Rudiger Höffer

**Affiliations:** 1 Faculty of Civil Engineering, University Ss. Cyril and Methodius, Skopje 1000, Macedonia; simona.bogoevska@gf.ukim.edu.mk (S.B.); dumova@gf.ukim.edu.mk (E.D.-J.); 2 Department of Civil, Environmental and Geomatic Engineering, ETH, Zürich CH-8093, Switzerland; mspyridonakos@gmail.com; 3 Department of Civil and Environmental Engineering, Ruhr-University Bochum, Bochum 44801, Germany; ruediger.hoeffer@ruhr-uni-bochum.de

**Keywords:** wind turbines, data-driven framework, uncertainty propagation, operational spectrum, time varying autoregressive moving average (TV-ARMA) models, polynomial chaos expansion (PCE)

## Abstract

The complex dynamics of operational wind turbine (WT) structures challenges the applicability of existing structural health monitoring (SHM) strategies for condition assessment. At the center of Europe’s renewable energy strategic planning, WT systems call for implementation of strategies that may describe the WT behavior in its complete operational spectrum. The framework proposed in this paper relies on the symbiotic treatment of acting environmental/operational variables and the monitored vibration response of the structure. The approach aims at accurate simulation of the temporal variability characterizing the WT dynamics, and subsequently at the tracking of the evolution of this variability in a longer-term horizon. The bi-component analysis tool is applied on long-term data, collected as part of continuous monitoring campaigns on two actual operating WT structures located in different sites in Germany. The obtained data-driven structural models verify the potential of the proposed strategy for development of an automated SHM diagnostic tool.

## 1. Introduction

The complexity related to the interacting subsystems of WT structures (namely the rotating blades, moving yaw mechanism, and pitch angle changes) and the alternating aerodynamic loads redefining the operational regime, result in a complex vibrating system necessitating adoption of efficient monitoring and diagnostic methods. The understanding of the intricate behavior of operating WT systems has preoccupied the research community since as early as the 1990s, resulting in development of operational modal analysis (OMA) methods, when in 1988 output-only testing was utilized for the first time on the 110 m high Éole turbine [1]. Although directly related with the birth of the nowadays widely implemented OMA techniques, the assessment of WT infrastructure has still remained a multidisciplinary, rather challenging task. Moreover, with Europe’s strategic planning currently focusing on renewable energy management, WTs are resurging into the spotlight for both the industrial and research community [2]. In this context, modern WT structures have to fulfil the growing demands for higher productivity and reduced downtime, which in turn calls for improved and automated SHM strategies, ensuring early-stage damage detection and structural diagnostics, reliability in power supply, as well as optimal operation and maintenance [3,4,5,6]. 

The difficulties related to assessment of operating WTs may be attributed among others sources to limited knowledge of the loading conditions (aeroelastic effects and rotor harmonics), the complexity of the multi-component WT system, varying operational regimes and environmental factors, as well as the typical uncertainties relating to incomplete and imperfect sensor data, modeling errors, complex and unique to the location soil-structure interaction effects. 

Considering OMA methods applied on onshore operating WT structures, several studies based on short-term measurement campaigns successfully presented different concepts and novel measurement techniques [7,8,9]. In more recent works implementation of long-term strategies for tracking variations in identified modal parameters has been reported, thus shifting the focus towards automated SHM schemes and interlinked fatigue assessment strategies [10,11,12,13,14,15]. In [10] Devriendt et al. present an automated identification algorithm that combines an OMA tool (the poly-reference least squares complex frequency-domain estimator or covariance-driven stochastic subspace identification method) with a double clustering approach (hierarchical and fuzzy clustering algorithms). The proposed method is validated via successful tracking of minor changes in the dynamic behavior of an offshore wind turbine on data continuously collected during a two-week interval, for idling, i.e., parked, condition of the structure. Iliopoulos et al. [11] propose and validate a response estimation methodology for acceleration predictions at arbitrary points of an offshore WT structure via fusion of a finite element model (FEM) of the structure, estimated OMA-based modal parameters and a limited number of measured acceleration time histories. In the work of Maes et al. [12], a full-scale verification of three response estimation algorithms for the prediction of dynamic strains along the tower of an offshore monopile wind turbine is presented. The proposed hybrid filtering approaches combine a system model, derived from an updated FEM of the structure (based on OMA identification of the parked system), and a limited set of acceleration, and combined acceleration and strain measurements. The fatigue assessment strategy is verified for parked and rotating conditions of the structure. Male and Lourens in [13] illustrated a strategy for real-time monitoring of WT fatigue accumulation by means of a joint input-state estimator. The method comprises of measuring operational vibrations at selected locations along a WT tower and strains assessment at unmeasured locations on the WT lattice support structure. The method is verified on artificial signals generated via a full-order FEM, whereas input forces and states are estimated on erroneous design model.

A significant point to keep in mind however is that the OMA class of methods is developed for implementation with time invariant systems [16], while more refined schemes pertaining to non-stationary systems do exist but are less frequently applied onto civil systems [17,18]. However, the major challenge for delivering an efficient performance-based long-term framework lies precisely in the time varying nature of WT structures, linked to the changing operational regimes and varying environmental agents, as well as in the misinterpretation of this variability [19]. The latter may result in false alarms hindering effective operation of associated damage detection and control intervention. For addressing the aforementioned challenge research studies in this field are mainly based on two general approaches. The first approach relies on filtering out the influence of environmental factors from estimated performance indicators, and comprises models which employ measured environmental/operational variables [15,20] or models which are solely based on structural response estimates [21]. The alternative approach lies in incorporation of the measured environmental variables into models of measured vibration response in the form of extracted performance indicators, e.g., modal parameters [22,23,24,25,26]. 

Related to the first concept of eliminating benign variations from estimated structural features, several works dedicated to operating wind turbine (OWT) structures have recently emerged. In order to compensate for the environmental variability, Weijtjens et al. in [15] subtracted a (non)-linear regression model from estimated resonant frequencies of an operating offshore WT. However, the operational variability of the WT dynamics is tackled via adoption of a case-by-case modal tracking SHM algorithm. A similar strategy of fitting multivariate linear and dynamic regression models is applied in Oliveira et al. [20] on a 2MW onshore WT. In the recent work of Hu et al. [21], the authors extracted a structural health index for a prototype of an operating 5MW WT, by removing temperature effects from selected natural frequency estimates based on a principal component analysis method. 

Novel strategies integrating both structural response data and influencing agents within mathematical models have proven successful in several recent studies. Addressing the non-stationarity in measured response, Spiridonakos et al. in [22] employed time varying autoregressive models to capture the short-term dynamics, and a polynomial chaos expansion (PCE) tool for capturing the influence of operational and environmental agents, in tracking the performance of an actual operating WT tower located in Lübbenau, Germany. The use of PCE for constructing metamodels of structural response that are adept in incorporating external influences in a long-term scale was initially introduced and successfully applied for the purpose of damage detection of the benchmark SHM project of the Z24-bridge in Switzerland by Spiridonakos and Chatzi in [23]. In a preliminary investigation, Bogoevska et al. in [24,25] examined the capacity of this framework for effective reduction of the input set dimension facilitating an implementation of the proposed approach in an automated fashion. It is also worth mentioning that a similar approach, this time relying on random coefficient (RC) linear parameter varying (LPV) AR models has appeared in the work of Avendano et al. in [18,26]. In [26] the RC-LPV-AR methodology is assessed on simulations of an operating wind turbine blade under different damage scenarios. The study confirms the potential of data-driven methodologies in the accurate modeling of the non-stationary and uncertain vibration response of operating WT blades, albeit exclusively demonstrated on simulation data. Indeed, automated strategies have so far mostly been demonstrated in either a laboratory setting or on numerical simulations [27,28,29]. 

The research study presented herein employs actual field data, from operating turbines, in order to extend the PCE-AR tool, developed by the authoring team [22,23,24,25], highlighting two main aspects: the robustness of the strategy, as well as the high potential of the proposed method for automated structural health assessment. The first aspect is demonstrated via implementation on two separate operating WT structures located in Germany for an extended period of monitored structural responses and larger set of influencing agents. This is accomplished by a bi-component tool, which combines the parametric smoothness priors time varying autoregressive moving average (SP-TARMA) method, for identifying structural performance indicators (short-term framework), with a PCE probabilistic model, for quantifying the uncertainty in the identified structural performance indicators (long-term framework). Towards the development of an autonomous diagnostic tool, capable of tracking and diagnosing structural condition during the complete WT life-cycle, a twelve-month tracking of an estimated PCE-SPTARMA diagnostic index is further demonstrated. In this context, as a preliminary step, the correlation between alerting values of the obtained diagnostic index and novel fluctuations in measured operating conditions is verified by means of outlier analysis. 

The proposed combined PCE-SPTARMA approach, in contrast to strategies incorporating commonly applied OMA methods, intrinsically captures the time variability of the system dynamics and delivers a holistic model, alleviating mode-by-mode or case-by-case modeling, for long-term tracking of the structural behavior. Furthermore, the results of the presented study demonstrate the effectiveness and high potential of the proposed method for automated condition assessment of large real world structures, operating in a wide range of conditions.

## 2. The Bi-Component Framework

The novel approach proposed herein relies on observation of the time-variability of WT structures in a dual-reference time-frame, namely: (a) the short-term scale, relating to periodic fluctuations induced by the rotational nature of components of the WT system itself as well as the nature of the input loads, and (b) the long-term scale, in which phenomena associated to constantly changing loading and environmental conditions perturb structural behavior. 

In satisfying the first goal, time-varying autoregressive moving average (TARMA) models deliver a suitable tool, capable of tracking the short-term variability of the structure. This parametric modeling technique proves adept in capturing the periodic variability and non-stationary dynamics of the operating structure, primarily attributed to: (i) forces linked to the environmental conditions, namely aerodynamic loads which originate from the linear increase in the vertical wind profile in the rotor-swept area, translated into cyclic load changes on the rotating rotor, and (ii) forces related to the operating conditions, i.e., gravitational forces whose direction relative to the blade is shifted during rotation, causing alternating periodic loading conditions. 

**Figure 1:**
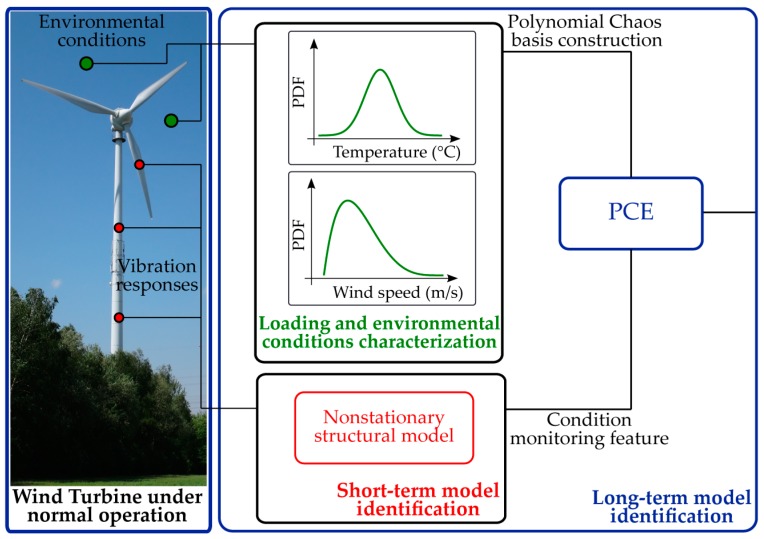
Schematic overview of the proposed bi-component monitoring strategy.

The long-term front is on the other hand focused on delivering the big picture, and therefore aims at accurate description of the WT dynamics over the complete operating spectrum of the structure. The typical stimuli linked to the long-term structural variability are changing within the range of hours, or even minutes and are usually related to changes in the wind flow profile, stochastic loads caused by wind turbulence, as well as variation of environmental conditions. For the purpose of modeling the evolution of the system response as a function of varying acting loads and operational conditions, the TARMA model is combined with the PCE method. The PCE model builds an expansion of a random output variable, e.g., an extracted TARMA model feature, on polynomial basis functions, which are orthogonal with respect to the probability density of the system’s random inputs, e.g., monitored environmental/loading parameters. The synergy between the two models enables a holistic assessment of a structure interacting with its environment. A schematic overview of the proposed strategy is presented in Figure 1. 

**Figure 2:**
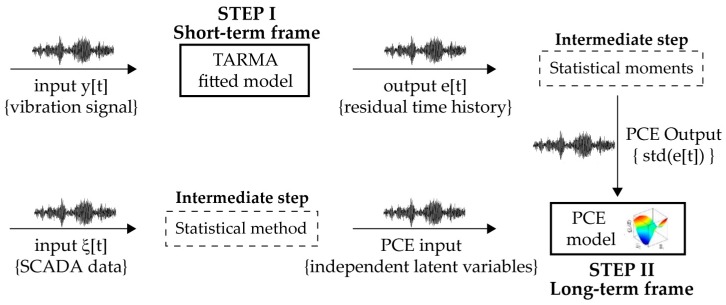
Steps of the proposed bi-component monitoring framework.

Considering for example a WT structure in regular operation, under the influence of ambient loading and changing environmental factors, a selected output feature (statistical moments of model residual) from the TARMA fitted model (step I) could be expanded on an appropriately constructed PC basis. By proper utilization of the TARMA model residuals (output parameter) and of the experimentally estimated operational and environmental variables (input parameter), the PCE model is finally constructed (step II). This coupled strategy provides the means for quantifying the uncertainty of WT dynamics due to randomness of the operational and environmental influencing factors. Furthermore, it delivers performance indicators in a standardized manner, which in turn contribute to development of a sophisticated damage detection strategy, where false fault alarms are timely recognized. An overview of the involved steps is presented in Figure 2. The following sections summarize the theoretical background underlying these steps. For more detailed information on the applied models, the interested reader is referred to [30,31,32,33,34,35,36,37,38,39,40].

### 2.1. Step I: Short-Term Framework

#### 2.1.1. The Model Structure

For a non-stationary signal y[t] the TARMA model, provides a compact parameterized formulation given in Equation (1), [30]. Similar to the descriptive nature of its stationary counterpart (i.e., the ARMA model), the TARMA model describes a regressive form of the present output y[t] on its past realizations y[t−1],…,y[t−n], taking into account modeling error and time-variability of the AR/MA parameters of the model:

(1) $$y\left[t\right]+\overset{n_{a}}{\underset{i=1}{\sum }}a_{i}\left[t\right]\cdot y\left[t-i\right]=e\left[t\right]+\overset{n_{c}}{\underset{i=1}{\sum }}c_{i}\left[t\right]\cdot e\left[t-i\right]\;e\left[t\right]∼NID\left(0,\sigma_{e}^{2}\left[t\right]\right)$$

where t designates discrete time (with i=1,2,…,N ) of the observed nonstationary signal y[t], e[t] is the residual sequence, i.e., the unmodeled part of the signal, assumed to be normally identically distributed with zero mean and time-varying variance $e\left[t\right]\sim NID\left(0,\sigma_{e}^{2}\left[t\right]\right)$, and $a_{i}\left[t\right]$, $c_{i}\left[t\right]$ the time-varying AR and MA parameters respectively, of AR, MA orders $n_{a},n_{c}$.

In general, the identification of a specific TARMA model comprises two steps: (i) proper selection of the model structure (the $n_{a}/n_{c}$ order for AR and MA parameters); (ii) estimation of the AR/MA parameters and the innovations variance $\sigma_{e}^{2}\left[t\right]$. However, for the specific subclass of smoothness-priors time-varying autoregressive moving average (SP-TARMA) models, the structure selection problem is further expanded to the selection of the smoothness constraints’ order κ. The order κ is directly related to an additionally imposed stochastic structure upon the AR and MA parameters. In order to constrain their time evolution, the SP-TARMA model enhances Equation (1) with the following set of supplementary stochastic difference equations, [30]:

(2) $${\left(1-B\right)}^{k}\;a_{i}\left[t\right]=w_{a_{i}}\left[t\right]\;w_{a_{i}}\left[t\right]∼NID\left(0,\sigma_{w_{a}}^{2}\left[t\right]\right)$$

(3) $${\left(1-B\right)}^{k}\;c_{i}\left[t\right]=w_{c_{i}}\left[t\right]\;w_{c_{i}}\left[t\right]∼NID\left(0,\sigma_{w_{c}}^{2}\left[t\right]\right)$$

where B is the backshift operator $\left(B^{k}x\left[t\right]=x\left[t-k\right]\right)$, κ designates the difference equation order, and $w_{i}\left[t\right]$ is a zero-mean, Gaussian sequences with time-dependent variance, uncorrelated, mutually uncorrelated and also uncrosscorrelated with e[t].

Thus, the full SP-TARMA model may be completely described by: (i) a model for the system response y[t], Equation (1), and (ii) a model which controls the time evolution of the AR and MA parameters of the first model, Equations (2) and (3). The selected AR parameter order $n_{a}$ represents the memory of the model and defines the number of past values of the signal, which are to be included into Equation (1). On the other hand, the stochastic smoothness constraints reflect our prior knowledge regarding the evolution of the underlying dynamics. The chosen order of the stochastic difference equations κ introduces the number of past values of AR/MA parameters to be incorporated in the stochastic model of the constraints, and thus determines the behavior of the time varying AR/MA coefficients. For example for order κ=1, the stochastic difference equations represent the random walk model, while for κ=2 each approximated AR/MA parameter locally follows a straight line and is distributed as a Gaussian white noise sequence [31]. The time evolution smoothness of the AR/MA parameters is furthermore influenced, in a reciprocal sense, by the variance $\sigma_{w_{i}}^{2}\left[t\right]$. Therefore, a third user selected parameter, the residual variance ratio $v=\sigma_{w}^{2}\left[t\right]/\sigma_{e}^{2}\left[t\right]$, controls the equivalent memory of the estimation algorithm between the two limit values: v→0, which implies a locally deterministic (polynomial) parameter evolution, and ν→∞ which implies no structure on parameter evolution [30]. An optimal value for ν may be achieved by means of statistical approaches, such as minimization of the AIC (Akaike information criterion) or the BIC (Bayesian information criterion). Both criteria employ a penalty term that controls the increase of the model order, thus ensuring adequate modeling precision without overfitting the N-sample-long modeled signal $y^{N}$, Equations (4) and (5):

(4) $$AIC=-2\cdot lnℒ\left(y^{N}\right)+2\cdot d$$

(5) $$BIC=-lnℒ\left(y^{N}\right)+\frac{lnN}{2}\cdot d$$

where ℒ(·) is the model likelihood, *N* the number of signal samples, and *d* is the number of independently estimated model parameters [30].

#### 2.1.2. Model Parameter Estimation

We reformulate the general (κ-th order) smoothness constraints equations, Equations (2) and (3), in the following form:

(6) z[t]=F·z[t−1]+G·w[t]

where:

(7) $$z\left[t\right]={\left[a_{1}\left[t\right]\ldots a_{n_{a}}\left[t\right]c_{1}\left[t\right]\ldots c_{n_{c}}\left[t\right]\vdots \cdots \vdots \;a_{1}\left[t-k+1\right]\ldots \;a_{n_{a}}\left[t-k+1\right]c_{1}\left[t-k+1\right]\ldots \;c_{n_{c}}\left[t-k+1\right]\right]}_{k\cdot \left(n_{a}+n_{c}\right)\times 1}^{T}$$

(8) $$w\left[t\right]={\left[w_{a1}\left[t\right]\;w_{a2}\left[t\right]\ldots w_{an_{a}}\left[t\right]\vdots \;w_{c1}\left[t\right]\;w_{c2}\left[t\right]\ldots w_{cn_{c}}\left[t\right]\right]}_{(n_{a}+n_{c})\times 1}^{T}$$

and F,G are matrices of the following forms (depending upon the value of κ):

(9) $$\begin{array}{lll} \kappa =1: & F≜I_{n_{a}+n_{c}}, & G≜I_{n_{a}+n_{c}},\\ \kappa =2: & F≜\left[\begin{array}{cc} 2\cdot I_{n_{a}+n_{c}} & -I_{n_{a}+n_{c}}\\ I_{n_{a}+n_{c}} & 0_{n_{a}+n_{c}} \end{array}\right], & G≜\left[\begin{array}{c} I_{n_{a}+n_{c}}\\ 0_{n_{a}+n_{c}} \end{array}\right],\\ \kappa =3: & F≜\left[\begin{array}{ccc} 3\cdot I_{n_{a}+n_{c}} & -3\cdot I_{n_{a}+n_{c}} & I_{n_{a}+n_{c}}\\ I_{n_{a}+n_{c}} & 0_{n_{a}+n_{c}} & 0_{n_{a}+n_{c}}\\ 0_{n_{a}+n_{c}} & I_{n_{a}+n_{c}} & 0_{n_{a}+n_{c}} \end{array}\right], & G≜\left[\begin{array}{c} I_{n_{a}+n_{c}}\\ 0_{n_{a}+n_{c}}\\ 0_{n_{a}+n_{c}} \end{array}\right], \end{array}$$

and so on,

$(I_{n}\;and\;O_{n}$ designate the n×n dimensional identity and zero matrices, respectively.)

The TARMA ($n_{a},n_{c}$) representation of Equation (1) may then be expressed as:

(10) $$y\left[t\right]=h^{T}\left[t,z^{t-1}\right]\cdot z\left[t\right]+e\left[t\right]$$

with:

(11) $$h\left[t,z^{t-1}\right]={\left[-y\left[t-1\right]\ldots -y\left[t-n_{a}\right]\vdots \;e\left[t-1,z^{t-1}\right]\ldots e\left[t-n_{c},z^{t-n_{c}}\right]\vdots \;0\ldots 0\right]}_{k\cdot \left(n_{a}+n_{c}\right)\times 1}^{T}$$

$z^{t}$ designates a vector containing all state vectors z[t] up to time t.

Hence, the SP-TARMA ($n_{a},n_{c}$) model may be completely expressed in state-space form, Equations (6) and (10). However, due to the unknown residual sequence $e\left[t,z^{t}\right]$ in $h\left[t,z^{t-1}\right],$ the full SP-TARMA case leads to a nonlinear state estimation problem. For this reason, i.e., for the recursive estimation of the state vector z[t], an Extended Least Squares (ELS)-like algorithm is employed. This leads to replacement of the theoretical prediction errors $e\left[t,z^{t}\right]$ in Equation (11) by their posterior estimates $\hat{e}\left[t,t\right]$, [30]:

(12) $$h\left[t\right]={\left[-y\left[t-1\right]\ldots -y\left[t-n_{a}\right]\vdots \;\hat{e}\left[t-1,t-1\right]\ldots \hat{e}\left[t-n_{c},t-n_{c}\right]\vdots \;0\ldots 0\right]}_{k\cdot \left(n_{a}+n_{c}\right)\times 1}^{T}$$

(13) $$\hat{e}\left[t|t\right]=y\left[t\right]-h^{T}\left[t\right]\cdot \hat{z}[t|t]$$

**Figure 3:**
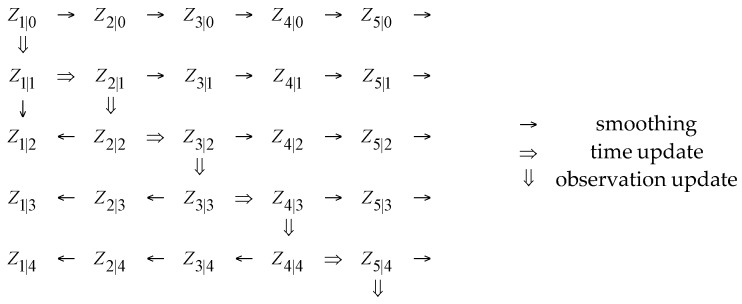
Schematic overview of the KF scheme (adapted from [32]).

Then, for a given and time-invariant order κ and residual variance ratio ν, the estimation of the state-space model may be achieved via the ordinary Kalman Filter (KF) scheme (Figure 3). The innovations (one-step-ahead prediction error) variance, $\sigma_{e}^{2}\left[t\right]$, may be estimated via a window of certain length, centered at time instant *t*, which slides over the prediction error (residual) sequence (more details and a summary of the normalized version of the KF method is provided in [30]). 

#### 2.1.3. SP-TARMA Based Dynamic Analysis

When the best-fit SP-TARMA model is identified, the time-varying dynamics of the structure may be recovered by the frozen-time spectrum [30]:

(14) $$S\left(\omega ,t\right)={\left|\frac{1+\sum_{i=1}^{n_{c}}c_{i}\left[t\right]e^{-j\omega T_{s}i}}{1+\sum_{i=1}^{n_{a}}a_{i}\left[t\right]e^{-j\omega T_{s}i}}\right|}^{2}\sigma_{e}^{2}\left[t\right]$$

where ω is the frequency in rad/sec, $T_{s}$ the sampling period in s, and j the imaginary unit. The frozen-time natural frequency $\omega_{ni}\left[t\right]$ and damping ratio $\zeta_{i}\left[t\right]$ of the system are related to the $\lambda_{i}\left[t\right]$ discrete-time ‘frozen’ pole of the TARMA model’s frozen frequency response function:

(15) $$\omega_{ni}\left[t\right]=\frac{\left|\ln\lambda_{i}\left[t\right]\right|}{T_{s}}\left(rad/sec\right)\;\mathrm{and}\;\zeta_{i}\left[t\right]=-\cos\left(\arg\left(ln\lambda_{i}\left[t\right]\right)\right)$$

### 2.2. Step II: Long-Term Framework (Modeling Uncertainty)

The time domain identification method described in Section 2.1, accounting for the non-stationarity of the WT system, is combined with a PCE tool in order to deliver a SHM framework capable of describing the comprehensive structural dynamics, in a long-term scale.

In contrast to the local representative nature and high computational cost of the well-known Monte Carlo collocation method and other similar sampling techniques, the PCE non-sampling approach is focused on constructing a functional dependence of the model output on the set of independent random variables (RVs) (following a certain probability law) that parametrizes the input data, usually expressed in terms of a series [33]. The polynomial chaos (PC) approach draws its origins in the modeling of stochastic processes via Gaussian RVs by employment of Hermite polynomials [34]. Later on, Cameron and Martin [35] demonstrated that for any arbitrary stochastic process with finite variance, the PC expansion converges in the ${ℒ}_{2}$ sense. The latter was generalized by Xiu et al. [36] to different continuous and discrete distributions using orthogonal polynomials of the so called Askey-scheme [37]. 

The main concept underlying the PCE framework is to represent a random quantity by means of expansion, comprising functions of random variables multiplied with deterministic coefficients. More precisely, PCE modeling generates an expansion of a random output variable on polynomial chaos basis functions, which are orthonormal with respect to the probability space of the system’s random inputs. For a system S featuring M random input parameters represented by independent random variables $\left\{\xi_{1},\ldots ,\xi_{M}\right\}$, gathered in a random vector Ξ of prescribed joint Probability Density Function (PDF) $p_{\Xi }\left(\xi \right)$, the system output, denoted by Y=S(Ξ), will also be random [38]. Provided that Y has finite variance, it can be expressed as follows:

(16) $$Y=S\left(\Xi \right)=\underset{d\in N^{M}}{\sum }\theta_{d}\varphi_{d}\left(\Xi \right)$$

where $\theta_{d}$ are unknown deterministic coefficients of projection, d is the vector of multi-indices of the multivariate polynomial basis (PC functions) $\varphi_{d}\left(\Xi \right)$, orthonormal to $p_{\Xi }\left(\xi \right)$. The PC polynomials are chosen so that the weights w(x), which ensure the orthogonal property.

$\langle \varphi_{i},\varphi_{j}\rangle =\int_{x_{1}}^{x_{2}}\varphi_{i}\left(x\right)\varphi_{j}\left(x\right)w\left(x\right)dx=h_{j}\delta_{ij}\;\left(\mathrm{with}\;\delta_{ij}=1\;\mathrm{if}\;i=j\;\mathrm{and}\;0\;\mathrm{otherwise}\right)$, resemble the PDF of the random variables. Several well-known families of orthogonal polynomials can be associated to a specific PDF, a more detailed overview may be found in [36]. 

The multivariate polynomials $\varphi_{d}\left(\Xi \right)$ used for constructing the PC model (for each term j of the PC series expansion) are obtained by tensor products of the corresponding univariate functions:

(17) $$\varphi_{d_{j}}\left(\Xi \right)=\overset{M}{\underset{m=1}{\prod }}\varphi_{d_{j,m}}^{1}\left(\Xi \right)$$

where *M* is the number of random input variables.

For ensuring timely computation, the functional series terms Equation (16) must be truncated to a finite number. The usual approach is the selection of the multivariate polynomial basis with total maximum degree $\left|d_{j}\right|=\sum_{m=1}^{M}d_{j,m}\leq P\;\forall \;j$. In this case the dimensionality of the functional subspace, i.e., the total number of series terms, equals:

(18) $$T=\frac{\left(M+P\right)!}{M!P!}$$

where P is the maximum total degree of the multivariate polynomials. The truncated PCE model of Equation (16) to the first T terms yields a finite number of deterministic coefficients and the parameter vector $\theta_{d}$ may be estimated by solving Equation (16) in a least squares sense [38]. 

**Figure 4:**
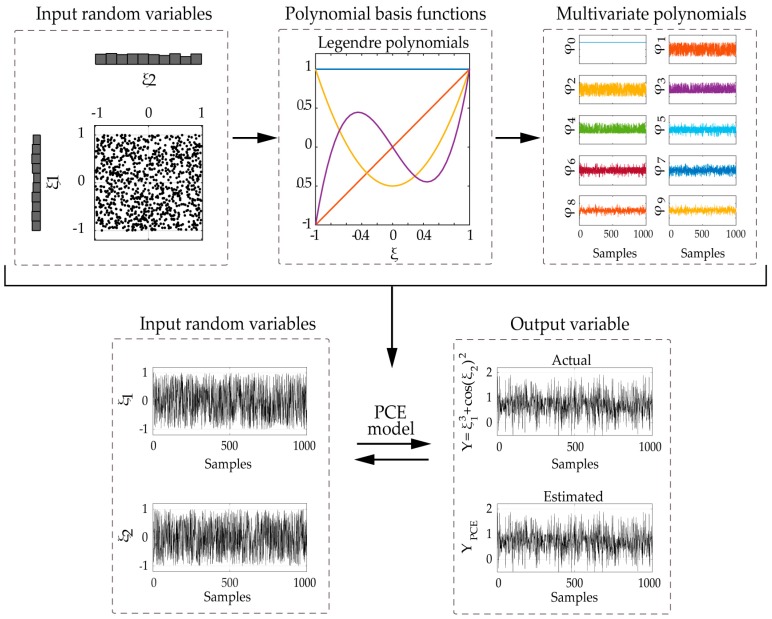
Schematic overview of the PCE method: example of two independent input random variables $\xi_{1},\xi_{2}$ with uniform pdf and a system output variable $Y=\xi_{1}^{3}+\cos{\left(\xi_{2}\right)}^{2}$.

For illustrating the construction of the PCE model, let us consider the simple case of a 2D-PCE model for two independent input random variables $\xi_{1},\xi_{2}$. By employing the first four univariate Legendre polynomials as PC basis functions for a selected total degree P = 3, we may develop the truncated PC expansion of Equation (19) (total number of terms T = 10, single index notation used). The construction of the 2D polynomials is presented in more detail in Table 1, and a schematic overview of the complete method is presented in Figure 4.

(19) $$\tilde{Y}\equiv \overset{9}{\underset{j=0}{\sum }}\theta_{j}\varphi_{j}=\theta_{0}+\theta_{1}\xi_{1}+\theta_{2}\xi_{2}+\theta_{3}\frac{1}{2}\left(3\xi_{1}^{2}-1\right)+\theta_{4}\xi_{1}\xi_{2}+\theta_{5}\frac{1}{2}\left(3\xi_{2}^{2}-1\right)+\theta_{6}\frac{1}{2}\left(5\xi_{1}^{3}-3\xi_{1}\right)+\theta_{7}\frac{1}{2}\left(3\xi_{1}^{2}-1\right)\xi_{2}+\theta_{8}\frac{1}{2}\left(3\xi_{2}^{2}-1\right)\xi_{1}+\theta_{9}\frac{1}{2}\left(5\xi_{2}^{3}-3\xi_{2}\right)$$

It is worth mentioning that in case of subpar performance of the standard PCE due to the rather limiting assumption of prescribed families of input distributions, an arbitrary or adaptive PCE scheme could serve as viable alternatives [33,38,39]. Unlike the classical approach, which utilizes a fixed form of PC basis functions, these variants employ PC functions adapted to the specific input variable characteristics.

#### Probabilistic Approximation of Random Variables

The prerequisite of independent input parameters, necessary for efficient PCE modeling, is here ensured by utilization of a statistical technique capable of inferring independent (latent) variables that are intermixed within observed data. Independent Component Analysis (ICA) is a computational approach that in contrast to Principal Component Analysis is concerned with the higher order statistics of the observable data, i.e., minimization of mutual information. ICA eventually aims at identifying non-Gaussian and mutually independent components [40], which may then be fed into the PCE component of the proposed SHM framework. 

The basic concept of ICA is in fact to describe how an observed random vector x is generated by a process of mixing unobservable (latent) variables. More specifically, if we consider n observed linear mixtures of n independent components $s_{i}$ (i=1,…,n) the following relation is assumed [40]:

(20) $$x_{j}=a_{j,1}s_{1}+a_{j,2}s_{2}+\ldots +a_{j,n}s_{n}\;\mathrm{for}\;j=1,\ldots ,n$$

or by using vector-matrix notation:

(21) x=As

with x designating the random observed variables vector, s the vector of unobserved independent variables and *A* the unknown mixing matrix:

$$A=\left[\begin{array}{ccc} a_{1,1} & \cdots & a_{1,n}\\ \vdots & \ddots & \vdots \\ a_{n,1} & \cdots & a_{n,n} \end{array}\right]$$

Via inversion of the mixing matrix A, the vector of the independent latent variables s becomes:

(22) $$s=A^{-1}x=Wx$$

ICA employs nonlinear optimization for estimating the mixing matrix *A*, which maximizes the non-Gaussianity of each one of the latent variables $w^{T}x$ (with w designating a column vector of matrix W). The adopted measure of non-Gaussianity, may be based on kurtosis, negentropy, and others [40]. A concise overview of the method, as well as a comprehensive flowchart of the ICA algorithm is presented in [23]. 

## 3. Steps of Application—Case Studies

### 3.1. Description of the Monitored Structures

The previously described bi-component SHM framework was initially implemented for tracking of the performance of an actual operating WT tower by utilizing 10 min long acceleration datasets, recorded every half hour for 29 days (from 18 December 2013 till 15 January 2014) [22]. Within this study the robustness of the strategy is further tested over a more extended time frame and a broader set of input parameters for the case of two operating WT structures, situated in different sites. 

**Figure 5:**
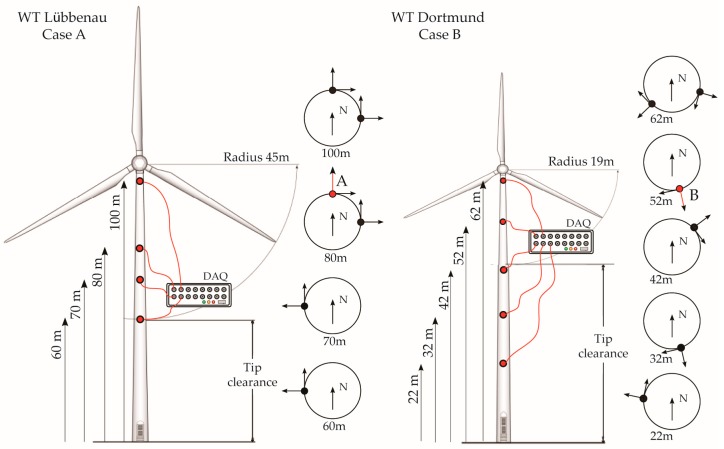
Schematic overview of the experimental setup (case A left and case B right).

The first case (in the further text: case A) is a 2MW WT which is part of a wind farm located in Lübbenau, Germany, [22], where access is obtained through collaboration with Repower Wind Lübbenau GmbH. The vibration response of the WT is measured via triaxial accelerometers (STM LIS344ALH MEMS sensors) at four distinct locations of the WT tower (Figure 5). The output-only vibration data is available in 10-min-long dataset records for each hour from 3 March 2015 till 6 May 2015, a total of 1388 datasets, measured at a 1600 Hz sampling rate. The SCADA data for the same time period are available in a 10-min average format. 

The second structure under study (case B) is a 0.5MW WT erected in 1997, located in the vicinity of Dortmund, Germany [41], where access is obtained through collaboration with Dortmunder Energie- und Wasserversorgung GmbH (DEW21). The acceleration response of the WT is measured by triaxial accelerometers (PCB-3713D1FD3G MEMS sensors) at five positions along the shaft (Figure 5). The vibration response as well as the SCADA data are sampled with the same frequency of 100 Hz. The implementation of the proposed SHM strategy for this case is presented for acceleration records of four complete months of continuously monitored data (May to August 2010).

### 3.2. Operational Modal Analysis for Parked Conditions

**Figure 6:**
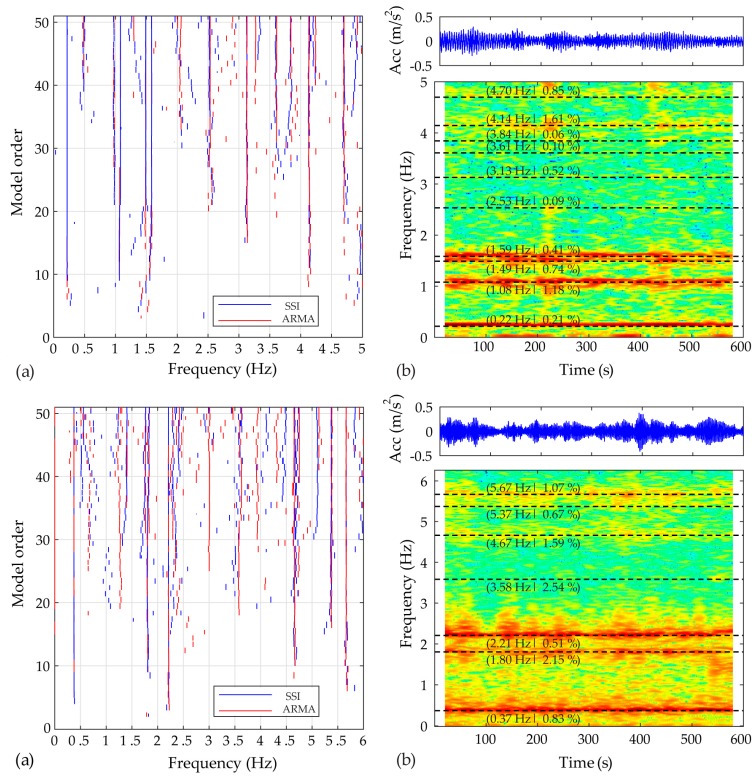
Dynamics of the parked WTs (Case A up and Case B bottom): (**a**) Stabilization plot for the stationary ARMA and SSI methods (model orders from 2 to 50); (**b**) Spectrogram and estimated natural frequencies and damping ratios based on an ARMA(32,32) model for Case A and ARMA(18,18) model for Case B (For interpretation of the references to color , the reader is referred to the web version of this article).

The initial identification of modal properties for both structures was carried out on datasets corresponding to parked conditions. For this purpose, the stationary ARMA method (prediction-error based) was applied for acceleration response signals measured at 80 m (Case A) and 52 m (Case B) height (directions marked as A and B in Figure 5). In Figure 6 the estimated stabilization diagrams for ARMA model orders between 2 and 50 are presented, together with results of the stochastic subspace identification method, based on the canonical variate algorithm for comparing evaluation. The ARMA estimated natural frequencies, for damping ratios less than 5%, for selected model orders equal to 32 and 18, for case A and B respectively, are summarized in this figure as well. The spectrograms (short time Fourier transform; Hamming data window; NFFT = 512; overlap 98%), appended in the background of the same plot, clearly demonstrate the stationarity of identified frequencies within the explored time frames.

### 3.3. Short-Term Framework Implementation

**Figure 7:**
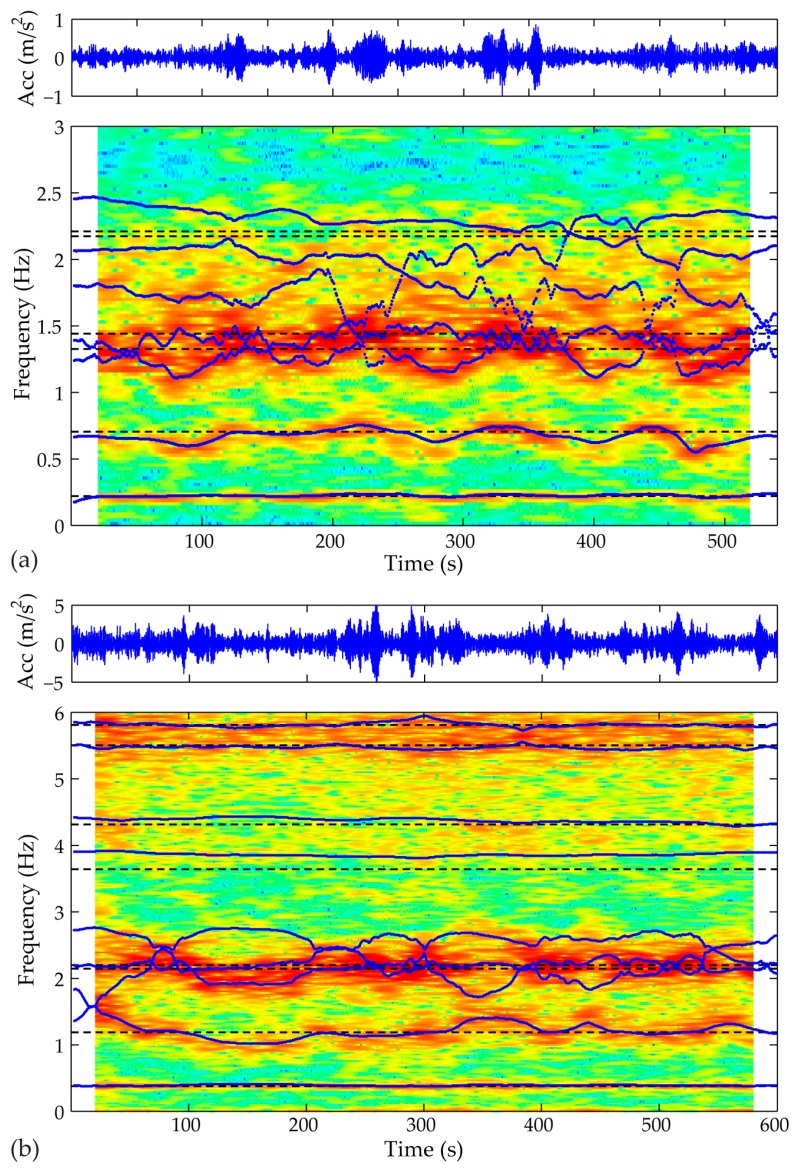
Dynamics of the WTs under normal operation: Stationary ARMA ($n_{a},n_{c}$) versus nonstationary SP-TARMA ($n_{a},n_{c}$) model estimates with spectrogram in the background (Short Time Fourier Transform; Hamming data window; NFFT = 512; overlap 98%). (**a**) Case A: ($n_{a},n_{c}$) = (32, 32); (**b**) Case B: ($n_{a},n_{c}$) = (18, 18).

In contrast to response that remains close to stationary, where the standard OMA techniques are commonly employed, the complex dynamic system of operating WT structures underlines the necessity for utilizing more efficient time-sensitive tools. The non-stationary dynamics of an operational WT structure can be clearly observed in Figure 7 by the spectrograms of a signal recorded at 2:00 p.m. 29 March 2015 (case A) and at 1:00 p.m. 31 June 2010 (case B). The SP-TARMA model (solid lines) is contrasted to the corresponding ARMA model (gray lines) and compared to the spectrograms in the background. As observed the SP-TARMA model is able to track the evolution of the time varying frequencies. The accuracy is different depending on the observed frequency. This precision is regulated by the residual variance ratio, which is directly linked to the evolution of the AR/MA coefficients, but therefore only indirectly linked to the evolution of the individual frequencies (Equation (15)). In separate work the optimal tuning of this parameter will be sought. However, we here demonstrate that this does not pose an issue for construction of the desired performance indicator. An alternate approach would be to filter the signal and separately treat the different frequency components, however there is a point to avoiding such practices as we here try to develop a framework that is minimally invasive with respect to the monitored signal. 

The same figure reveals additional frequencies fluctuating around 0.7 Hz (Case A) and 1.2 Hz (Case B), which match to the 3P harmonics of the rotating WT structures (for the corresponding time frames mean RPM equal 15 and 24, for Case A and B respectively). It is worth noting that in contrast to strategies where existent harmonics interfere the process of structural identification, the short-term tool framework delivers a robust performance indicator, without the necessity of eliminating alternating dynamic effects introduced by the operating WT components. Moreover, the ability of the proposed tool for incorporated tracking of existing harmonics may further facilitate the detection of potential rotor related faults (i.e., unbalanced masses).

For the short-term framework implementation, the acceleration time histories for both cases were low-pass filtered and down-sampled to 12.5 Hz, cutoff frequency at 5 Hz (Case A) and 6 Hz (Case B), observed as 10-min long data sets. This results in total of 1388 data sets for Case A, and 17 706 data sets for case B. The acceleration records are utilized with their originally measured orientation. However, the underlying directional dependency is incorporated via the continuously measured WT nacelle orientation (yaw angle), which is used as an input parameter in the long-term modeling framework of the next step.

**Figure 8:**
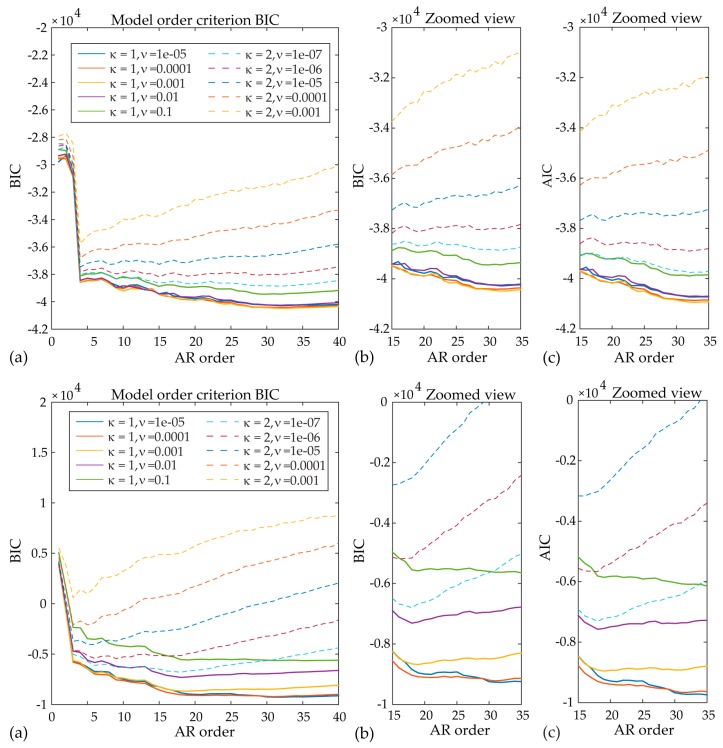
Statistical criterion for model order selection (Case A top and Case B bottom): (**a**) Bayesian statistical criterion; (**b**) Bayesian statistical criterion zoomed-in view; (**c**) Akaike statistical criterion zoomed-in view (For interpretation of the references to color, the reader is referred to the web version of this article).

**Figure 9:**
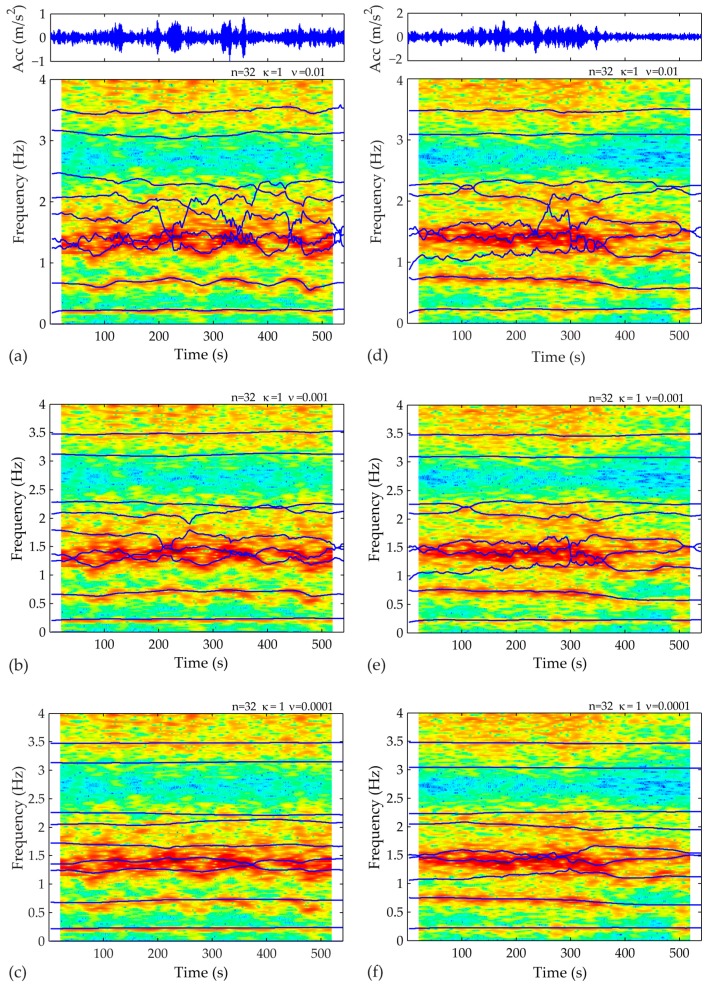
SP-TARMA (n = 32, k = 1) model tuning for Case A by varying the value of the ratio of model residuals. First sample of a 10-min acceleration signal fitted with: (**a**) ν = 0.01; (**b**) ν = 0.001; (**c**) ν = 0.0001; Second sample of a 10-min acceleration signal fitted with: (**d**) ν = 0.01; (**e**) ν = 0.001; (**f**) ν = 0.0001.

**Figure 10:**
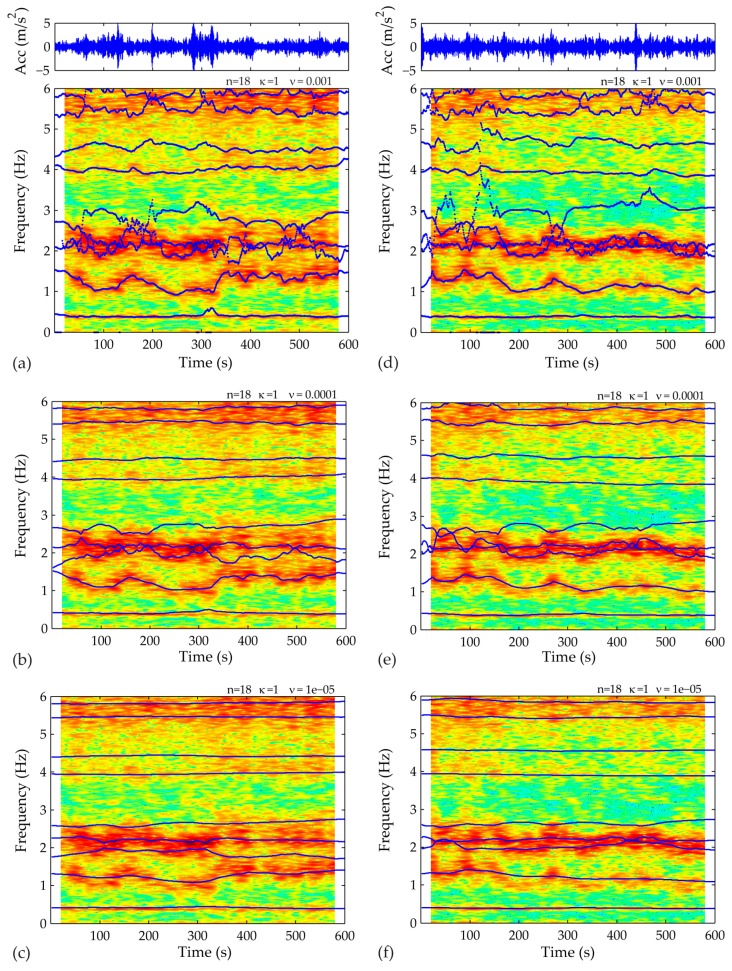
SP-TARMA (n = 18, k = 1) model tuning for Case B by varying the value of the ratio of model residuals. First sample of a 10-min acceleration signal fitted with: (**a**) ν = 0.001; (**b**) ν = 0.0001; (**c**) ν = 0.00001; Second sample of a 10-min acceleration signal fitted with: (**d**) ν = 0.001; (**e**) ν = 0.0001; (**f**) ν = 0.00001.

After preprocessing the acceleration records, the first step of the proposed framework lies in fitting an appropriate SP-TARMA model to the actual 10-min structural response signals. Toward this end, the user-defined parameters of the SP-TARMA model (i.e., the model order n, the smoothness constraint order κ and the residual variance ratio ν) have to be properly tuned. The plots of the Bayesian and Akaike statistical criterion for model order selection are presented in Figure 8. The zoomed views (Figure 8b,c) indicate the range of values that may lead to adequate modelling of the nonstationary signal, without overfitting it. The BIC and AIC deliver similar results for both case studies. For Case A, however, the values minimizing the criteria functions do not adequately track the variation of the frequency at the vicinity of 0.7 Hz (Figure 9). Thus, in ensuring an automated operation of the short-term tool the values n = 32, κ = 1, ν = 0.01, are selected. On the other hand, the inspection of the SP-TARMA estimates for Case B (Figure 10) reveals a good agreement with the parameter values that minimize the penalty functions (n = 18, κ = 1, ν = 0.0001). 

### 3.4. Long-Term Framework Application

**Figure 11:**
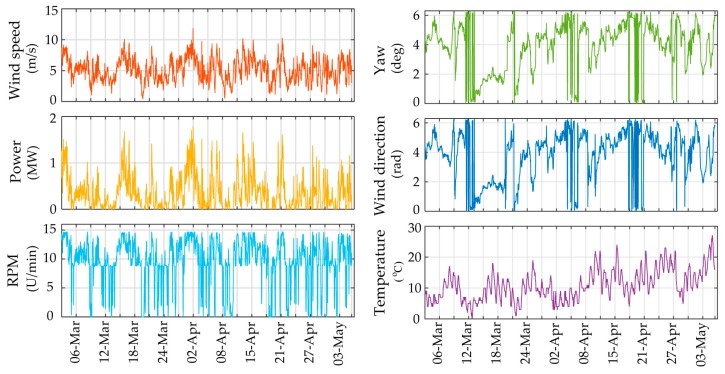
Time history plots of selected PCE input variables for 1388 datasets (Case A).

**Figure 12:**
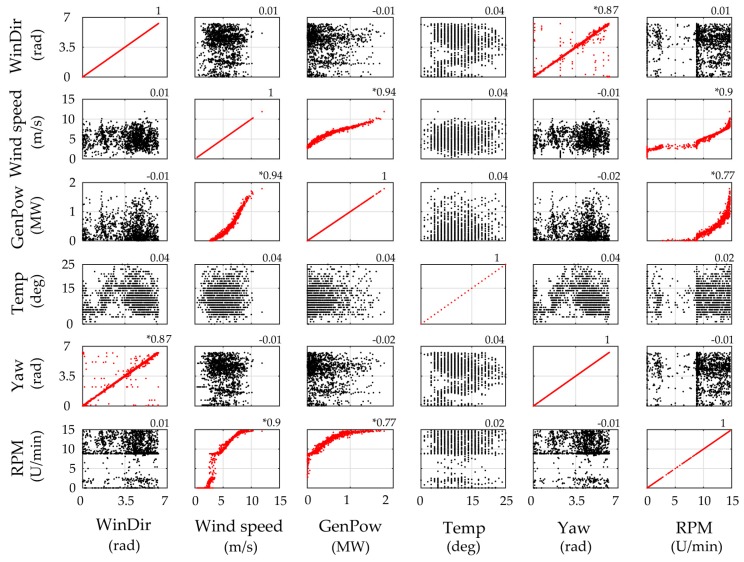
Scatter plots of selected PCE input variables and estimated correlation values (Case A).

**Figure 13:**
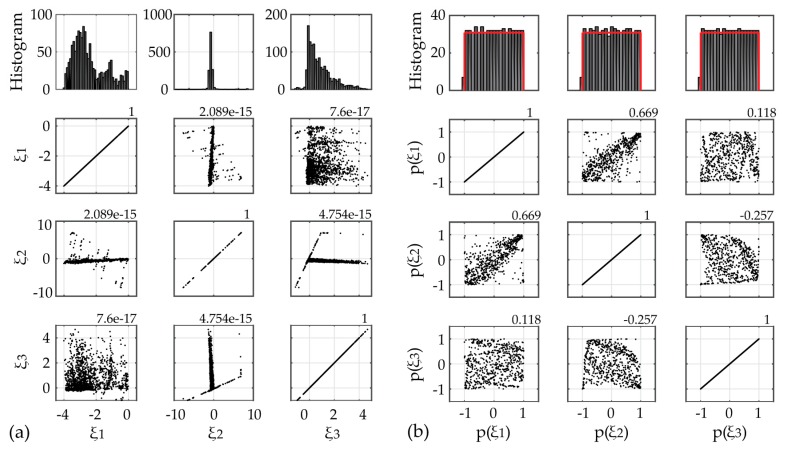
Input data for Case A: (**a**) Scatter plots of ICA-based input variables and estimated correlation values, histograms of the latent variables (upper row); (**b**) Scatter plots of the random vector Ξ of prescribed uniform PDF $p_{\Xi }\left(\xi \right)$, histograms of the random vector Ξ values (upper row).

For utilizing the long-term framework, input variables corresponding to operational and environmental measured data need to be selected. In Figure 11 the 10 min averages of the six selected SCADA parameters for Case A are plotted. The correlation plots for each pair of chosen output variables is presented in Figure 12. It is apparent that several pairs may be assumed as correlated with correlation larger than 0.6 (marked with asterisk). In order to transform the input data to independent variables, thus satisfying the PCE method requirement, the ICA method is herein applied. The minimum required number of ICA variables fully describing the input data variance is revealed via inspection of the eigenvalues of the covariance matrices of the selected SCADA variables. For the first application case study, the corresponding three ICA-derived latent variables are presented in Figure 13a. 

For the purpose of constructing the random vector Ξ of prescribed joint PDFs $p_{\Xi }\left(\xi \right)$, the ICA estimates are further transformed into uniformly distributed variables via use of the non-parametrically estimated cumulative distribution functions, Figure 13b.

**Figure 14:**
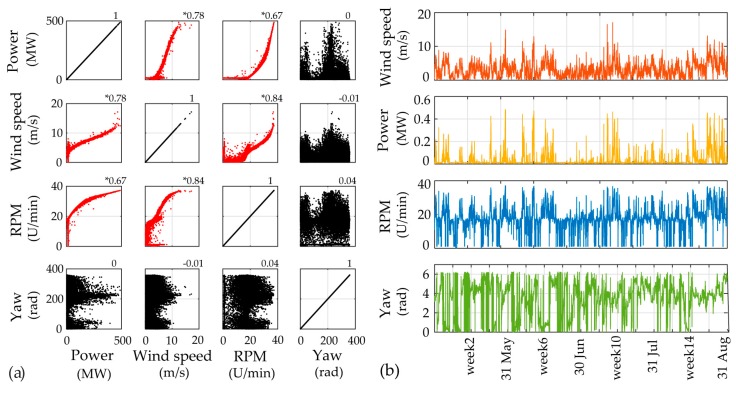
(**a**) Scatter plots of selected PCE input variables and estimated correlation values; (**b**) Time history plots of the selected PCE input variables for 17 706 datasets (Case B).

**Figure 15:**
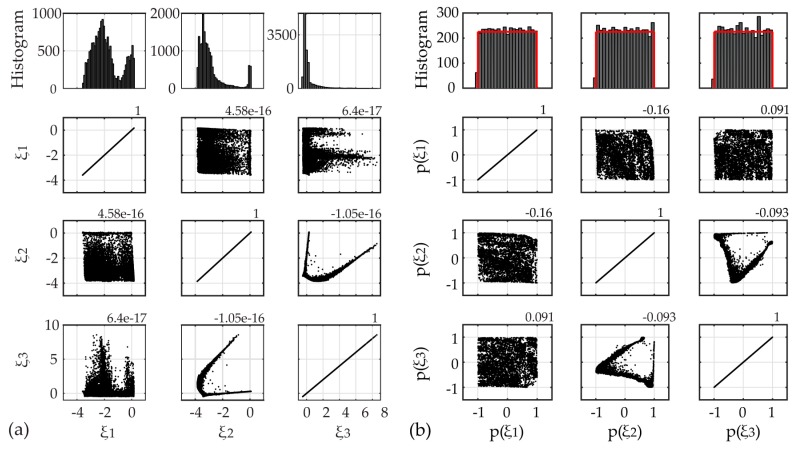
Input data for Case B: (**a**) Scatter plots of ICA-based input variables and estimated correlation values, histograms of the latent variables (upper row); (**b**) Scatter plots of the random vector Ξ of prescribed uniform PDF $p_{\Xi }\left(\xi \right)$, histograms of the random vector Ξ values (upper row).

For the second case study the long-term framework is utilized for an expanded time frame of four full months. The time histories of the four selected input variables as well as their correlation values are plotted in Figure 14. The plots of the ICA-derived latent variables and scatter plots of the random vector Ξ of prescribed uniform PDF, after the twofold transformation of the input parameters to independent and uniform variables, are presented in Figure 15.

As a last step, the SP-TARMA output variables and the PDFs of the measured operational input data are fed into the PCE (long-term) framework. In accordance with the uniform PDFs of the input data, the Legendre polynomials are selected as the PC functional basis. The maximum polynomial order is selected equal to three (case A) and five (case B). Further increasing the maximum order does not significantly improve the accuracy of the expansion. The standard deviation (std) of the SP-TARMA residuals for the 10 min intervals are selected as the PCE output parameter. 

**Figure 16:**
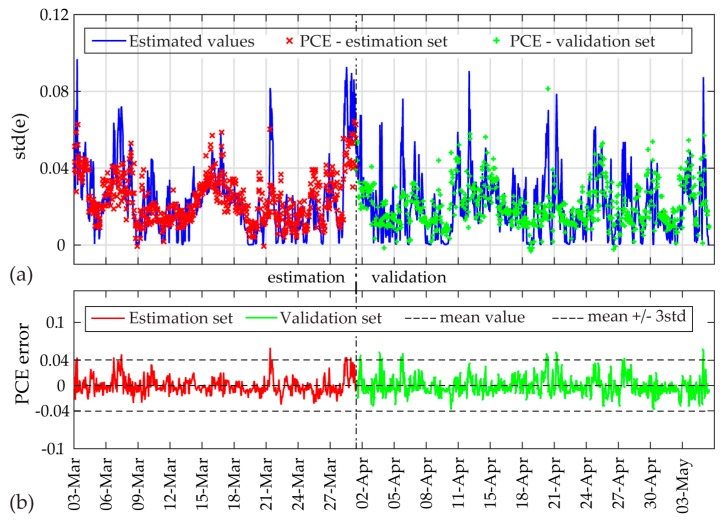
PCE estimates for Case A: (**a**) std of SP-TARMA(32,32,1,0.01) model residual and PCE model expansion values; (**b**) The PCE errors along with 99.7% confidence intervals (calculated for estimation set).

**Figure 17:**
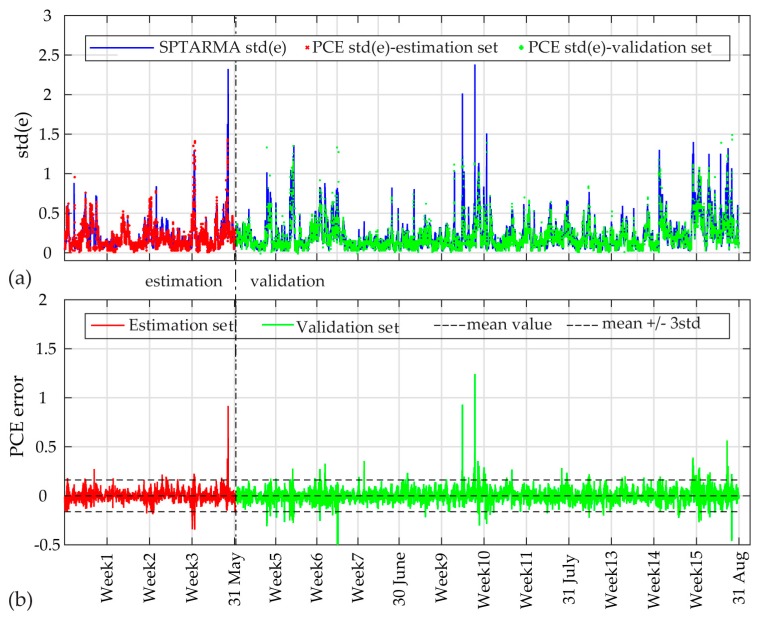
PCE estimates for Case B: (**a**) std of SP-TARMA(18,18,1,0.0001) model residual and PCE model expansion values; (**b**) The PCE errors along with 99.7% confidence intervals (calculated for estimation set).

The standard deviation (std) of the residuals for each dataset and the PCE model estimates are plotted in Figure 16 and Figure 17 for both cases. For case A, the total of 1388 data sets for the two month period, are divided into an estimation (45%) and validation (55%) period. The PCE errors are plotted in the same figure, along with the corresponding 99.7% confidence intervals calculated for the fitted Gaussian distribution of the estimation set errors. For case B, the total monitoring period of 4 months is divided into a one-month estimation period and a three-month validation period. As observed, the PCE model is capable of simulating the std(e) output variable with very good accuracy for both cases, and the model residual falls within the 99.7% confidence intervals for both sets. For the actual structures under study no damages were observed, with results verifying the applicability of the proposed framework for the continuous monitoring period of the two studied systems (summarized in Table 2).

## 4. PCE-TARMA Diagnostic Index

The SHM-based system diagnostics is built on training data from the baseline, or “healthy” structure under regular environmental and operational conditions. The obtained models are then implemented with newly acquired data, and deviations from the established normal conditions are detected as a novelty [28]. A robust diagnostic tool should be able to distinguish between true system changes and benign alarms, originating from new ranges of measured input data. In this case, instead of alarming for a potential maintenance intervention, model retraining should be carried out on the basis of the extended data set. 

**Figure 18:**
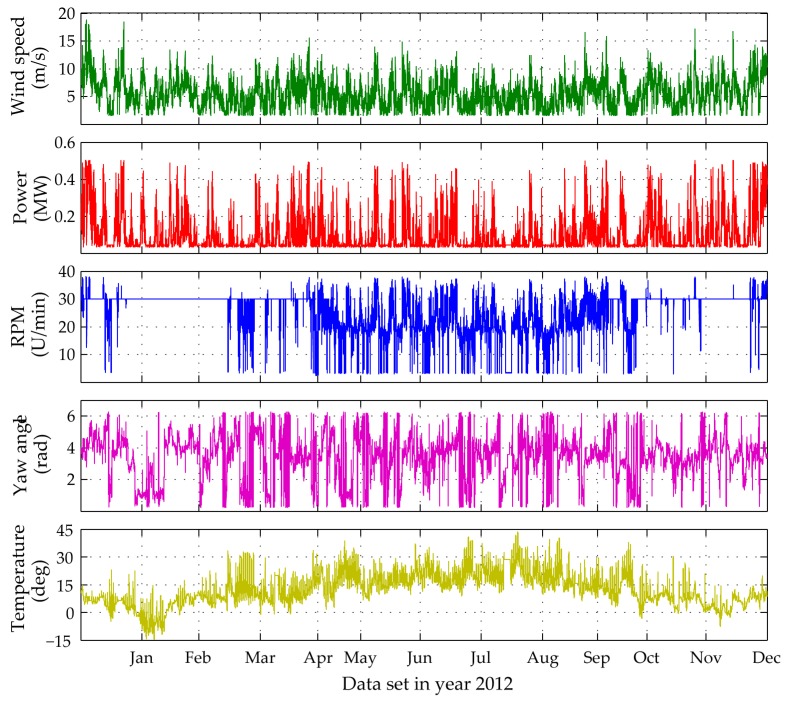
Time history plots of the selected PCE input variables for 49,956 datasets corresponding to complete year 2012.

In this context, the developed PCE-TARMA model responsiveness to varying environmental and operational parameters (EOP) is tested for two, three and four-month long training periods, with an input of five distinct SCADA variables (Figure 18). Based on the previously described framework in Section 3, the long-term tracking is extended to one complete year time-frame of monitored data for the operating Case B WT structure. The PCE-TARMA model is refitted with the model parameters summarized in Table 2, Section 3.4. During the twelve-month tracking period influencing agents characterized by seasonal variations would bear a significant impact. Therefore, in this setting the temperature measured at the outer side of the WT tower shaft (at 20 m height) is incorporated as a PCE model input as well. 

**Figure 19:**
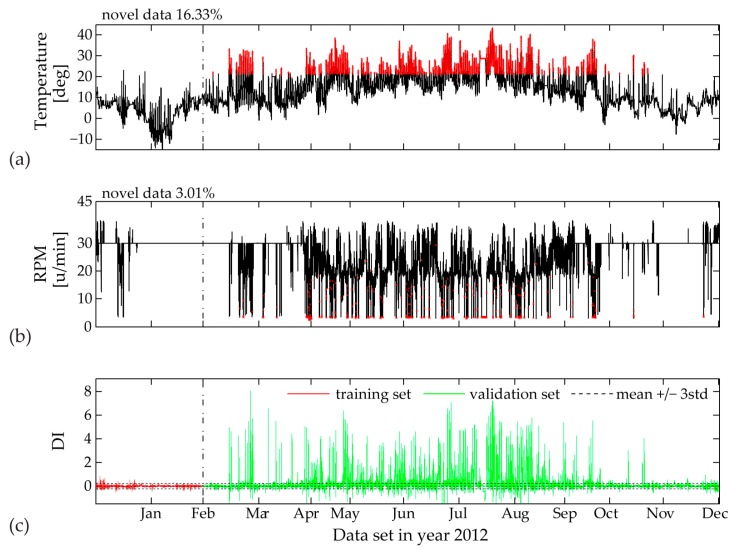
Two-month training set: (**a**) Identified novel data (red points) within time history of 10-min mean values of measured temperature; (**b**) Identified novel data (red points) within time history of 10-min mean values of measured RPM; (**c**) Diagnostic Index for the complete data sets of year 2012.

**Figure 20:**
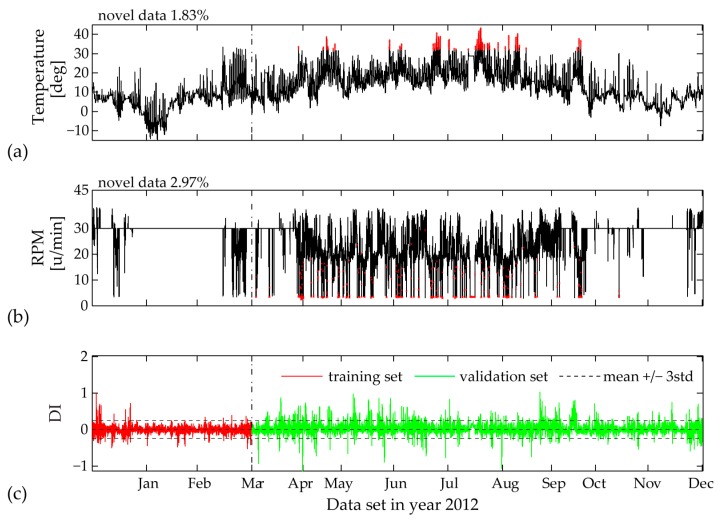
Three-month training set: (**a**) Identified novel data (red points) within time history of 10-min mean values of measured temperature; (**b**) Identified novel data (red points) within time history of 10-min mean values of measured RPM; (**c**) Diagnostic Index for the complete data sets of year 2012.

**Figure 21:**
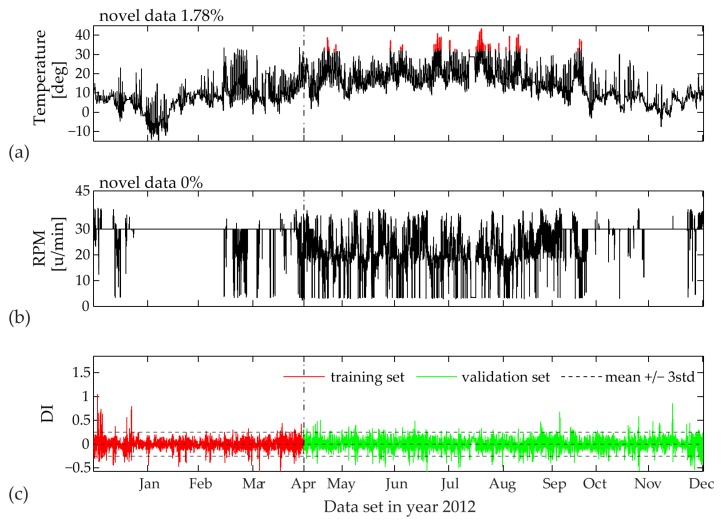
Four-month training set: (**a**) Identified novel data (red points) within time history of 10-min mean values of measured temperature; (**b**) Identified novel data (red points) within time history of 10-min mean values of measured RPM; (**c**) Diagnostic Index for the complete data sets of year 2012.

In Figure 19, Figure 20 and Figure 21 the validation sets of the estimated PCE residual demonstrate the workings of a consistent Diagnostic Index (DI) able to directly illustrate unfamiliar fluctuations in the EOP. Following statistical outlier analysis the index values which exceed the thresholds of ± 3std can be linked to new data ranges of measured influencing agents.

For this purpose, the well-known Mahalanobis Distance (MD) method is herein applied on the input data time histories. For a p-dimensional multivariate sample $x_{1},\ldots ,x_{n}$, the MD is defined as, [42]:

(23) $$MD_{i}=\sqrt{{\left(x_{i}-t_{tr}\right)}^{T}C_{tr}^{-1}\left(x_{i}-t_{tr}\right)}\;for\;i=1,\ldots ,n$$

where $t_{tr}$ is the arithmetic mean and $C_{tr}$ is the sample covariance matrix, both estimated for an input data set corresponding to a certain training period length. For normally distributed multivariate data, the MD values result approximately chi-square distributed with p degrees of freedom $\left(\chi_{p}^{2}\right)$, while the MD threshold is usually set as a certain quantile of $\chi_{p}^{2}$. However, an adaptive method that takes into account the actual empirical chi-square distribution function of the estimated MD (instead of a fixed quantile) is herein applied [42]. In this way thresholds (adjusted quantiles) are estimated for corresponding training sets for each of the five SCADA parameters. The $x_{i}$ samples from the validation sets which have MD beyond the predefined threshold are defined as novelties.

False triggering alarms due to incomplete training set are mostly related to input parameters characterized by unpredictable range of values, i.e., temperature measurements and wind velocity. In this case for a two-month training period, the univariate MD plot of the temperature time history shows the highest percentage of 16.33% novel data (Figure 19). The DI distribution pattern, plotted in the same figure, is clearly linked to the new upcoming values between months March and November. If we further increase the training set to a three-month period (Figure 20), the MD outlier percentage for measured temperature decreases. Correspondingly, the DI becomes significantly reduced, although not yet within the ± 3std limit values. In Figure 20, for the same three-month training period, an interesting observation is the contribution of the rotor rotation to novel data (2.97%), which is completely reduced to 0% after four months of model training (Figure 21). The reason for this occurrence is the relatively flat range of RPM values between mid-January and mid-March, most probably due to a sensor malfunction, which later on is repaired. 

The MD plots (Figure 20 and Figure 21) demonstrate that for a full incorporation of various fluctuations in the measured temperature, the training period should be even extended to eight months. Additional univariate MD analysis for the measured wind velocity, power production and yaw angle for the complete year 2012 reveals no new data points within the validation sets of these inputs, even for a training period lengths of one to two months (January and February). The estimated minimum PCE model training set lengths for capturing the complete scope of the separate SCADA variables is summarized in Table 3.

## 5. Future Work

Statistical pattern recognition concepts [28] represent a favorable detection approach for SHM data-driven diagnostic strategies based on measured responses of structures in a healthy, or unknown baseline condition. Recent studies [15,43] reported successful application of control chart based strategies for monitoring structural changes in WT systems. Dervilis et al. in [27] investigated the applicability of various neural networks techniques towards online accurate monitoring of WT blades under continuous fatigue loading. In the field of bridge monitoring, Dervilis et al. in [44] developed residual outlier maps which reveal different spatial manifestation of detected outliers related to changing environmental and operational conditions, versus structural damages. 

In this context, for completing the scope of the proposed tool, future work is targeted at testing the assessment capabilities of the PCE-SPTARMA method by expanding the validation data range to subsequent recorded years. Through long-term tracking of the estimated DI, typical operating regimes to specific structural behavioral patterns of the WT system will be interrelated. The final goal lies in development of an algorithm that separates benign pattern distortions, linked to EOP fluctuations, from malignant pattern alterations due to actual structural damage or system malfunction. Accordingly, the diagnostic tool triggers a model retraining or appropriate notification procedure.

## 6. Conclusions

Europe’s growing needs for energy savings have placed WT structures in focus, albeit the tackling of complexities of these structures has long occupied both the academic and industrial communities. At the same time, the recent technological developments in sensors and monitoring solutions motivate the enhancement of the currently adopted SCADA stream for WTs with information of structural nature, able to guide operators in the management of these assets. 

In materializing such a goal, it is important to account for the non-stationary response of operating WTs and the temporal variability of their identified modal parameters. In order to develop comprehensive dynamic models both issues need to be addressed. By merging environmental and operational variables with a time-varying model of vibrational response, the proposed bi-component tool serves as the first step towards automated condition assessment. 

Successful implementation of the devised strategy on two distinct operating WT structures in Germany for different settings, namely, different sites, turbine types, SCADA duration, and different influencing agents, verifies the robustness of the approach. Furthermore, the outcomes of the complete one-year tracking of the obtained diagnostic index demonstrate the potential of the proposed tool for incorporation within a holistic SHM damage detection framework, further extended via statistical pattern recognition methods, to be explored in a next step. Another interesting perspective lies in fusing the surrogate models developed as part of this diagnostic framework with a simulation-based design procedure, where simulations of the SP-TARMA model for diverse loadings may be used for predictions on processes such as fatigue, equivalent damage loads, etc.

## Figures and Tables

**Table 1:** Multivariate 2D Legendre polynomials for total polynomial order P = 3.

First Four 1D Legendre Polynomials $L_{j}\equiv \varphi_{d_{j}}^{1}$	Single Index *j*	Multi-Index *d*	$\overset{M}{\underset{m=1}{\prod }}\varphi_{d_{j,m}}^{1}\left(\Xi \right)$	
$L_{0}=1$	0	(0,0)	$L_{0}\;L_{0}$	$\varphi_{0}=1$
$L_{1}=x$	1	(1,0)	$L_{1}L_{0}$	$\varphi_{1}=\xi_{1}$
$L_{2}=0.5(3x^{2}-1)$	2	(0,1)	$L_{0}L_{1}$	$\varphi_{2}=\xi_{2}$
$L_{3}=0.5(5x^{3}-3x)$	3	(2,0)	$L_{2}L_{0}$	$\varphi_{3}=0.5\left(3\xi_{1}^{2}-1\right)$
	4	(1,1)	$L_{1}L_{1}$	$\varphi_{4}=\xi_{1}\xi_{2}$
	5	(0,2)	$L_{0}L_{2}$	$\varphi_{5}=0.5\left(3\xi_{2}^{2}-1\right)$
	6	(3,0)	$L_{3}L_{0}$	$\varphi_{6}=0.5\left(5\xi_{1}^{3}-3\xi_{1}\right)$
	7	(2,1)	$L_{2}L_{1}$	$\varphi_{7}=0.5\left(3\xi_{1}^{2}-1\right)\xi_{2}$
	8	(1,2)	$L_{1}L_{2}$	$\varphi_{8}=0.5\left(3\xi_{2}^{2}-1\right)\xi_{1}$
	9	(0,3)	$L_{0}L_{3}$	$\varphi_{9}=0.5\left(5\xi_{2}^{3}-3\xi_{2}\right)$

**Table 2:** Summary of the applied bi-component tool for the two case studies.

PCE-TARMA Tool	SP-TARMA (Short-Term Modeling)			PCE (Long-Term Modeling)		
	Input	Model	Output	Input	Model	Output
**Case A: 2MW WT** **Vibration response** 10-min. datasets, 1/h, Mar–May 2015 sampling at 1600 Hz **SCADA data** 10-min averages	- 1388 total datasets - filtered and down-sampled to 12.5 Hz	*n* = 32, κ = 1, ν = 0.01	residual time histories 10-min long	3 ICA-based variables from 6 SCADA variables	- order P = 3 - Legendre polynomials - estimation 45% of data - validation 55% of data	standard deviation of 10-min. long residual time histories (1388 values)
**Case B: 0.5MW WT** **Vibration response** 1 h datasets, cont., May–Aug 2010 sampling at 100 Hz **SCADA data** 1 h datasets sampling at 100 Hz	-17 706 total datasets -filtered and down-sampled to 12.5 Hz	n = 18, κ = 1, ν = 0.0001	residual time histories 10-min long	3 ICA-based variables from 4 SCADA variables	- order P = 5 - Legendre polynomials - estimation 25% of data - validation 75% of data	standard deviation of 10-min. long residual time histories (17,706 values)

**Table 3:** Minimum necessary training lengths for capturing the complete SCADA data value ranges.

Power							
Wind							
Yaw angle							
Rotor RPM							
Temperature							
Jan	Feb	Mar	Apr	May	Jun	Jul	Aug
